# Integrase-Defective Lentiviral Vector Is an Efficient Vaccine Platform for Cancer Immunotherapy

**DOI:** 10.3390/v13020355

**Published:** 2021-02-23

**Authors:** Valeria Morante, Martina Borghi, Iole Farina, Zuleika Michelini, Felicia Grasso, Alessandra Gallinaro, Serena Cecchetti, Antonio Di Virgilio, Andrea Canitano, Maria Franca Pirillo, Roberta Bona, Andrea Cara, Donatella Negri

**Affiliations:** 1 Department of Infectious Diseases, Istituto Superiore di Sanità, 00161 Rome, Italy; val.morante7@gmail.com (V.M.); martina.borghi@iss.it (M.B.); iole.farina1@gmail.com (I.F.); felicia.grasso@iss.it (F.G.); 2 National Center for Global Health, Istituto Superiore di Sanità, 00161 Rome, Italy; zuleika.michelini@iss.it (Z.M.); alessandra.gallinaro@iss.it (A.G.); andrea.canitano@iss.it (A.C.); mariafranca.pirillo@iss.it (M.F.P.); roberta.bona@iss.it (R.B.); 3 Confocal Microscopy Unit NMR, Confocal Microscopy Area Core Facilities, Istituto Superiore di Sanità, 00161 Rome, Italy; serena.cecchetti@iss.it; 4 Center for Animal Research and Welfare, Istituto Superiore di Sanità, 00161 Rome, Italy; antonio.divirgilio@iss.it

**Keywords:** lentiviral vector: vaccine, immunotherapy, tumor, immune tolerance, tumor associated antigen

## Abstract

Integrase-defective lentiviral vectors (IDLVs) have been used as a safe and efficient delivery system in several immunization protocols in murine and non-human primate preclinical models as well as in recent clinical trials. In this work, we validated in preclinical murine models our vaccine platform based on IDLVs as delivery system for cancer immunotherapy. To evaluate the anti-tumor activity of our vaccine strategy we generated IDLV delivering ovalbumin (OVA) as a non-self-model antigen and TRP2 as a self-tumor associated antigen (TAA) of melanoma. Results demonstrated the ability of IDLVs to eradicate and/or controlling tumor growth after a single immunization in preventive and therapeutic approaches, using lymphoma and melanoma expressing OVA. Importantly, LV-TRP2 but not IDLV-TRP2 was able to break tolerance efficiently and prevent tumor growth of B16F10 melanoma cells. In order to improve the IDLV efficacy, the human homologue of murine TRP2 was used, showing the ability to break tolerance and control the tumor growth. These results validate the use of IDLV for cancer therapy.

## 1. Introduction

Cancer is the second leading cause of mortality worldwide, as reported by The World Health Organization (WHO) with 9.6 million deaths in 2018. A wide range of cancer immunotherapies have been developed and some of these have been approved in recent years, including preventive and therapeutic cancer vaccines [1,2], CAR (chimeric antigen receptor)-T cells [3,4] and immune checkpoint inhibitors [5,6].

In cancer immunology, the immunological tolerance represents a significant obstacle to be removed in order to obtain an efficient and comprehensive immune response against tumor antigens constituted by self-proteins (tumor associated antigens, TAAs). Different melanoma TAAs used in melanoma immunotherapy approaches include gp100, MART-1, and three enzymes associated with melanin synthesis, tyrosinase (tyr), tyrosinase related protein-1 and -2 (TRP1 and TRP2). These proteins have similar structures, but they are expressed by different genes and have different enzymatic activities. TRP1, or gp75, is a DHICA oxidase in melanin biosynthesis while TRP2 is a DOPAchrome tautomerase, they are highly expressed in melanosomes where the melanin synthesis takes place [7]. TRP2 is localized also in the trans-golgi network of melanoma cells, suggesting that it may be involved in other regulatory functions unrelated to melanogenesis pathway [8]. A recent paper suggests that TRP2 over-expression may be an important mediator of intrinsic drug resistance in melanoma cells [9]. The murine B16 cell line, a very aggressive tumor cell line widely used in preclinical models of tumor growth [10], expresses the murine TRP2 (mTRP2) which shares 80% homology with human TRP2 (hTRP2) at the protein level. Induction of TRP2-specific cytotoxic T lymphocytes (CTL) has already been demonstrated both in humans and mice [11,12]. Moreover, since TRP2 is located on plasma membrane, it can be considered as a target for ADCC activity [13,14].

DNA and viral vector-based vaccines (genetic vaccines) represent an effective method to generate an antigen-specific CD8+ T cell response by expressing the antigen of interest directly in target cells in vivo, in combination with activator signals of innate immunity [15,16]. Viral vectors are extensively used as delivery system of vaccine antigens both prophylactically, to prevent infections and therapeutically, to treat cancer and other diseases. Lentiviral vectors (LVs) have been used as vehicles for gene therapy or for vaccine applications demonstrating high efficiency in a variety of preclinical and clinical settings. The main concerning feature of LV involves its ability to integrate stably in the host genome [17,18] which, on the other hand, is crucial for gene therapy application based on stable transduction of proliferating cells [19,20]. However, since integration poses a risk of insertional mutagenesis, LVs use is limited in humans. Therefore, integrase-defective lentiviral vectors (IDLVs) have been developed [21]; they are unable to integrate in the host genome and produce unintegrated extrachromosomal DNA (E-DNA), which has been shown to be transcriptionally active. Unintegrated vector E-DNA is able to express viral proteins and to persist in vitro and in vivo in non-dividing cells [22,23]. IDLVs are currently under evaluation for gene therapy and vaccines in preclinical and clinical studies [24,25]. Our group demonstrated that a single immunization with IDLV pseudotyped with VSV.G envelope induced a strong and durable antigen specific immune response through the induction of polyfunctional CD8+ T cells as well as production of antibodies in mice and nonhuman primates [26,27,28]. We also showed that therapeutic vaccination with a single dose of IDLV delivering a non-oncogenic form of the Human papillomavirus (HPV) E7, was able to eradicate E7 positive tumors in mice [29]. Recent studies showed that a similar platform based on IDLV pseudotyped with a modified Sindbis virus envelope, designed to target dendritic cells in vivo via binding of CD209 and expressing the full-length human cancer-testis antigen NY-ESO-1, generated a significant clinical benefit after three intradermal doses in a phase I clinical trial in patients with advanced and metastatic synovial sarcoma expressing NY-ESO-1 [30]. 

The aim of this study is the validation of our vaccine platform based on IDLV pseudotyped with VSV.G as delivery system of different TAAs for cancer immunotherapy, in preclinical murine models. We performed in vitro and in vivo experiments in order to optimize the anti-tumor activity of our vaccine strategy, using model antigens such as ovalbumin (OVA) as non-self TAA in lymphoma and melanoma models and TRP2 as a self-antigen in the melanoma model. We demonstrated in different tumor models the ability of IDLV to control tumor growth after a single immunization in both preventive and therapeutic approaches.

## 2. Materials and Methods 

### 2.1. Cell Lines

Lenti-X 293T human embryonic kidney cell line was obtained from Clontech (Mountain View, CA, USA). Cells were maintained in Dulbecco’s modified Eagle’s medium, DMEM (Gibco Life Technologies Italia, Monza, Italy), supplemented with 10% fetal bovine serum FBS (Corning, Mediatech Inc., Manassas, VA, USA), and 100 units/mL penicillin/streptomycin/glutamine (PSG) (Gibco). The EG.7-OVA cell line (EG7, CRL-2113; American Type Culture Collection), a stable transfectant of the murine OVA-expressing EL4 thymoma, was maintained in RPMI, 10% FBS, 50 mM 2-mercaptoethanol (Sigma Chemicals, Co., St. Louis, MO, USA), and 0.4 mg/mL Geneticin. B16F10 cell line obtained from Interlab cell line collection (ICLC, Genoa, Italy) is a C57/BL6 syngeneic model of melanoma. Cells were maintained in DMEM supplemented with 10% FBS, 100 units/mL PSG, non-essential amino acids (Euroclone S.p.A, Milan, Italy), sodium pyruvate 1 mM (Euroclone, Milan, Italy). B16OVA cell line, provided by Claude Leclerc, Insitut Pasteur, Paris, France [31] was maintained in RPMI 1640 (Euroclone), supplemented with 10% FBS, 100 units/mL of penicillin/streptomycin/L-glutamine (Euroclone), 2 mg/mL Geneticin (Gibco), and 60 µg/mL hygromycin (Hoffmann-Roche, Basilea, Switzerland).

### 2.2. Construction and Production of IDLV

Schematic representation of plasmids used in this report is shown in Supplementary Figure S1. Lentiviral transfer vector pTY2CMV-GFPW and pTY2CMV-OVAW expressing GFP and OVA from internal CMV promoter were described previously [29,31]. Transfer vectors pTY2CMV-mTRP2W and pTY2CMV-hTRP2W were obtained by inserting the codon optimized mTRP2 and hTRP2 coding sequences (Twin Helix, Milan, Italy) into pTY2CMV-GFPW (using AgeI/EcoRV restriction sites) and pTY2CMV-OVAW (using AgeI/SalI restriction sites), respectively. To obtain the transfer vectors expressing mTRP2 fused to enhancer sequences, Calreticulin (CRT) and mouse or human major histocompatibility complex class II–associated invariant chain (mIi and hIi, respectively), CRT/hIi/mIi sequences were codon optimized and inserted into pUC57 plasmids (TwinHelix). CRT sequence was excised from pUC57-CRT plasmid with restriction enzymes XbaI/EcoRI, while mIi and hIi sequences were excised from pUC57 plasmids using NheI/EcoRI restriction enzymes. Purified CRT/hIi/mIi fragments were fused in frame with mTRP2 excised from pTY2CMV-mTRP2W using EcoRI/SalI restriction enzymes and inserted into NheI/SalI restricted pTY2CMV-OVAW, replacing OVA sequence and introducing the fusion protein, thus obtaining the transfer vectors pTY2CMV-CRT-mTRP2W, pTY2CMV-mIi-mTRP2W, and pTY2CMV-hIi-mTRP2W. The Integrase (IN) competent packaging plasmid pΔR8.2 was originally obtained from I. Verma (Salk Institute, La Jolla, CA, USA). The IN defective packaging plasmid pcHelp/IN-, containing a D116N mutation in the IN coding sequence, was originally obtained by Dr. Jacob Reiser, (NIH, Bethesda, MD, USA). Plasmid pMD.G, obtained from D. Trono (EPFL, Lausanne, Switzerland) produces the vesicular stomatitis virus envelope glycoprotein G (VSV.G), for pseudotyping the vector particles [32]. Recombinant IDLV were produced in Lenti-X cells as previously described [26]. Briefly, Lenti-X cells were transiently transfected on 10 cm Petri dishes using the Calcium Phosphate Profection Mammalian Transfection System (Promega Corporation, Madison, WI, USA). A total of 15 μg of plasmid DNA were used for each plate in a ratio 4:8:3 (transfer vector:packaging vector:VSV.G vector). After 48 h, cell culture supernatants were recovered, cleared from cellular debris, and passed through a 0.45 μM pore size filter (Millipore Corporation, Billerica, MA, USA). For viral concentration, vector-containing supernatants were ultracentrifuged (Beckman Coulter, Inc., Fullerton, CA, USA) on a 20% sucrose cushion (Sigma Chemical Co. St. Louis, MO, USA) and viral pellets were resuspended in phosphate-buffered saline (PBS, Gibco, Life Technologies Italia, Monza, Italy) and stored at −80 °C until use. Viral titers were evaluated by the reverse transcriptase (RT) activity assay as previously described [26].

### 2.3. Western Blotting

To verify the expression of the transgene, Lenti-X cells were seeded in 10 cm plates and transfected or transduced with plasmids or vectors expressing TRP2 and GFP at 37 °C in atmosphere containing 5% CO_2_. Thirty-six hours post-transfection or post-transduction supernatants and cells were collected. Equivalent amounts of cells, supernatants, and vector preparations were lysed in lysis buffer (20 mM HEPES, 50 mM NaCl, 10 mM EDTA, 2 mM EGTA, 0.5%, NP-40, 50 mM NaF, 1 mM orthovanadate, 1 mM PMSF, 5 mg/mL of aprotin, and 5 mg/mL of leupeptin). Proteins were separated on 12% SDS polyacrylamide gel and transferred to a nitrocellulose membrane (GE Healthcare, Chicago, IL, USA). B16F10 cells were used as positive controls for expression of TRP2. The filters were saturated overnight with 5% nonfat dry milk (NFDM) in PBST (PBS with 0.1% Tween 20) and then incubated with rabbit anti-TRP2 polyclonal antibody (ab74073, Abcam, Cambridge, UK) for 1 h at room temperature, followed by incubation for 1 h at room temperature with an anti-rabbit HRP-conjugated IgG (Sigma). The immunocomplexes were visualized using chemiluminescence ECL detection system (Luminata Crescendo Western HRP Substrate, Millipore).

### 2.4. In Vivo Experiments

Female C57BL/6 mice (6–8 weeks old) were purchased from Charles River (Calco, Como, Italy) and kept in pathogen-free condition in the animal facilities at the Istituto Superiore di Sanità (ISS, Rome, Italy). All animal studies were authorized by the Italian Ministry of Healthy after being approved by the Center for Animal Research and Welfare of ISS (Authorization n. 314/2015-PR, approval date 30 April 2015). All animal procedures were performed according to European Union guidelines and Italian legislation (Decreto Legislativo 4 March 2014, n. 26).

IDLV was administered intramuscularly (i.m.) in a maximum final volume 100 µL/leg, at doses indicated in the schedule of immunization described in detail for each experiment in the Results section. Mice were injected subcutaneously (s.c.) with 100 µL of PBS containing either 3.5 × 10^6^ E.G7-OVA, 2 × 10^5^ B16OVA or 5 × 10^4^ B16F10 cells per mouse, as specified for each experiment.

### 2.5. Tissue Processing

Mice were bled orbitally under metaphane-induced anesthesia, using sterile glass Pasteur pipette with heparin (Sigma) to collect whole blood. Plasma was separated by centrifugation at 2000 rpm for 10 min and stored at −80 °C. After addition of ammonium-chloride-potassium buffer (ACK) to lyse erythrocytes, cells were centrifuged at 1500 rpm for 10 min and then washed with PBS for 5 min. Cells were then suspended in appropriate volume of complete medium containing RPMI 1640, 10% FBS, 100 units/mL PSG, Pyruvate Sodium 1 mM, HEPES buffer 25 mM, 100 units/mL non-essential amino acids, and 50 mM 2-mercaptoethanol and counted. At sacrifice, spleen and tumor mass were harvested. Single-cell suspensions were prepared by mechanical disruption of spleens and passage through cell strainers (BD Pharmingen, San Jose, CA, USA) in the presence of 3 mL of ACK. Splenocytes were washed with 5 mL complete medium. After centrifugation at 1500 rpm for 10 min at 4 °C, cells were suspended in appropriate volume of complete medium and counted. The tumor mass was harvested and snap-frozen in Tissue-Tek OCT medium (VWR, Monroeville, PA, USA) in a liquid nitrogen (LN2)-cooled isopentane bath (Sigma, St. Louis, MO, USA).

### 2.6. IFNγ ELISpot Assay

The IFNγ ELISpot assay was performed using the BD ELISPOT kit reagents and protocol (BD Biosciences, San Jose, CA, USA). Briefly, 96-well plates (MAIPS4510 Millipore) were coated with purified anti-IFNγ antibody overnight and then blocked with complete medium. Single cell suspensions from spleen or blood were seeded at a density of 2.5 × 10^5^/well and stimulated overnight either with 2 µg/mL of the H-2Kb restricted OVA 8mer peptide (SIINFEKL) or TRP2 9mer peptide (SVYDFFVWL), as specific stimulation (PRIMM, Milan, Italy). Concanavalin A (5 µg/mL, Sigma Chemicals) was used as a positive control, while complete medium was used as negative control. Cells were then removed and the biotinylated anti-IFNγ antibody followed by streptavidin-HRP (Horse radish peroxidase) were added. After washes, the substrate 3-amino-9-ethylcarbazole (AEC) (Sigma Chemicals) was added for 15 min and the reaction was stopped with water. Spot Forming Cells (SFC) were counted using an ELISpot reader (A.EL.VIS, Hannover, Germany) and results expressed as specific SFC per 10^6^ cells. SFC number obtained in the medium-treated wells (background) was subtracted from SFC number found in peptide-treated wells. Samples were scored positive when a minimum of 50 specific SFC per 10^6^ cells and a fold ≥2 compared to the background were observed.

### 2.7. Measurement of Specific IgG Antibodies by ELISA

Plasma samples were tested for the presence of anti-OVA and anti-TRP2 IgG antibodies by a standard ELISA. Ninety-six well plates (Greiner bio-one, Frickenhausen, Germany) were coated with 0.5 μg/well of OVA (Sigma) or murine TRP2 proteins (MyBioSource, San Diego, CA, USA) overnight at 4 °C. After washing and blocking for 2 h with 200 μL of PBS containing 1% BSA (Sigma Chemicals), serial dilutions of plasma from individual mice were added to wells in duplicate and incubated for 2 h at room temperature. The plates were washed and biotin-conjugated goat anti-mouse IgG (Southern Biotech, Birmingham, AL, USA) was added to the wells for 2 h at room temperature. The plates were washed again before the addition of horse radish peroxidase (HRP)-conjugated streptavidin (AnaSpec, Fremont, CA, USA) for 30 min at room temperature. The antigen–antibody reaction was measured by using the 3.3,5.5-tetramethylbenzidine substrate (SurModics BioFX, Edina, MN, USA) and the reaction was stopped with 50 μL of H_2_SO_4_ 1 M. Endpoint titers were determined as the reciprocal of the highest dilution giving an absorbance value at least equal to threefold that of background (biological sample from naïve mice). For each group of immunization, results were expressed as mean titer ± standard error.

### 2.8. Intracellular Staining to Detect Cytokines Production

Splenocytes were seeded in U-bottom 96-well plates at 2 × 10^6^/well. Cells were cultured in the presence of GolgiPlug containing Brefeldin A (BD Biosciences) and Monensin (Biolegend, San Diego, CA, USA) to inhibit cytokine secretion, as well as Alexafluor 488 conjugates anti-mouse CD107a (Biolegend), as a marker of cytotoxic activity. Cells were then stimulated with OVA 8mer peptide (5 µg/mL) or left untreated, in the presence of anti-mouse CD28 mAb (BD Pharmingen) at 40 µg/mL. As a positive control, stimulation with Phorbol 12-myristate 13-acetate, PMA (10 ng/mL) (Sigma) as protein kinase C activator, in combination with Ionomycin (1 µg/mL) (Sigma), a calcium ionophore, was used. Cells were incubated 5 h at 37 °C and then left overnight at 4 °C. Intracellular cytokine staining was performed using Foxp3/Transcription Factor Staining Buffer Set (eBioscience, ThermoFisher, Waltham, MA, USA). Briefly, after blocking of Fc receptors by treatment with anti-mouse CD16/CD32 (BD Pharmigen) cells were stained with APC conjugated anti-mouse CD3 (Immunological Sciences) and PerCP-Cy5.5 anti-mouse CD8a (BD Pharmigen) for 20 min at 4 °C. Cells were washed, fixed following the steps of the protocol, and stained with PE conjugated anti-mouse IFNγ (BD Pharmigen) and PECy7-conjugated anti-mouse TNFα (BD Pharmigen) for 30 min at room temperature. Samples were washed and acquired using FACScanto (BD Biosciences) and analyzed using the FlowJo software (v10).

### 2.9. Confocal Laser Scanning Microscopy (CLSM) Analyses

Sections 8 µm thick were cut from OCT embedded tissue blocks with a Leica CM1850 UV cryostat (Leica Microsystems, Wetzlar, Germany) and placed on charged microscope slides for staining. Slides were blocked for 60 min using 10% FBS with 0.03% Triton X-100 (Thermo Fisher) in 1X PBS and then incubated with a rat anti-mouse CD3 antibody (Immunological Science, dilution 1:50) followed by goat anti-rat Alexa Fluor^®^ 594 (dilution 1:200, Life Technologies), or with anti-mouse MHC Class I antibody (Immunological Science, dilution 1:50) followed by goat anti-mouse Alexa Fluor^®^488 (dilution 1:200, Life Technologies), or with rabbit anti-OVA antibody (AB1225, Millipore, dilution 1:200) followed by goat anti-rabbit Alexa Fluor^®^488 (dilution 1:200, Life Technologies), or with rabbit anti-TRP2 antibody (Abcam, diluted 1:100), followed by goat anti-rabbit Alexa Fluor^®^488 (dilution 1:200, Life Technologies). B16F10 cells and Lenti-X were seeded on coverslips coated with 10 μg/mL poly-L-Lysine (Sigma-Aldrich) and placed into a 24-well cluster plates. Lenti-X cells were transfected with pTY2CMV-mTRP2W in a final volume of 100 µL/slide for 7 h. At 72 h post-transfection cells were fixed with 3% paraformaldehyde for 30 min at 4 °C. After a further washing with PBS, fixed cells were stored at 4 °C until analysis (within a month). To selectively detect TRP2 expression, Lenti-X and B16F10 cells were permeabilized with 0.5% Triton X-100 for 10 min at room temperature and then stained, with rabbit polyclonal anti-TRP2 antibody diluted 1:100, followed by goat anti-mouse Alexa Fluor^®^488 (dilution 1:200, Life Technologies). Coverslips were mounted with Vectashield^®^ antifade mounting medium containing DAPI (Vector Laboratories). CLSM observations were performed with a Zeiss LSM980 apparatus, equipped with a 40x oil objective, Airyscan2 and excitation spectral laser lines at 405, 488, 543, 594, and 647 nm. Image acquisition and processing was carried out using Zen Blue edition 3.1 (Zeiss, Oberkochen, Germany) and Adobe Photoshop CS5 software programs (Adobe Systems, San Jose, CA, USA). Cells and tissue sections stained only with the fluorochrome-conjugated secondary antibody were used to set up acquisition parameters. Signals from different fluorescent probes were taken in sequential scanning mode. Several fields of view (>200 cells) were analyzed for each labeling condition, and representative results are shown.

### 2.10. Statistical Analysis

Statistical analyses were performed using Prism software (GraphPad Software, Inc., La Jolla, CA, USA). The log-rank Mantel-Cox test was used to compare the survival distributions. Mann Whitney and unpaired T test were used to compare immune response, tumor growth, or cell phenotype among groups, as specified for each experiment in the Results section.

## 3. Results

### 3.1. A Single Immunization with IDLV-OVA in E.G7-OVA-Bearing Mice Eradicated the Large Tumor Mass and Generated a Protective Anti-Tumor Memory Response

**Figure 1:**
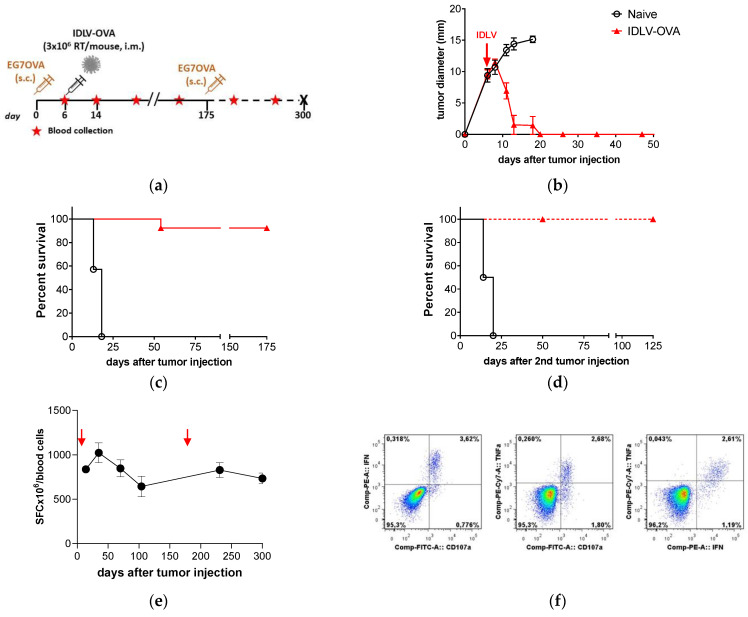
Therapeutic efficacy of integrase-defective lentiviral vectors (IDLV)-ovalbumin (OVA) vaccination in E.G7-OVA-bearing mice. (**a**) Scheme of the experiment: 14 C57BL/6 mice were inoculated s.c. with 3.5 × 10^6^ E.G7-OVA cells/mouse. Mice with 7–9 mm diameter tumor mass were vaccinated with 3 × 10^6^ RT/mouse of IDLV-OVA (*n* = 7) or left untreated (Naïve, *n* = 7). Tumor growth, survival, and immune response were monitored over time. Tumor-free mice were injected a second time with tumor cells at 175 days and monitored up to the end of the experiment (300 days). (**b**) Tumor growth after the first tumor injection is shown. Mice were sacrificed when the tumor diameter reached 15 mm or an ulceration of tumor was observed. (**c**,**d**) Kaplan–Meier survival curves are shown. Survival was monitored up to 175 days after the first tumor injection (Log-rank Mantel-Cox test) and up to 125 days from the second tumor injection (Log-rank Mantel-Cox test). (**e**) Kinetics of OVA-specific T cell response in IDLV-OVA vaccinated mice, after E.G7-OVA injection (red arrows). Blood cells were collected at the indicated time points and stimulated with the H-2Kb restricted OVA 8mer peptide (SIINFEKL). Data are expressed as specific spot forming cells (SFC) per 10^6^ cells. Error bars indicate the standard deviation among the animals from the same group. (**f**) Polyfunctional OVA-specific CD8+ T cells. Vaccinated and tumor-free mice were sacrificed at 300 days and splenocytes were used to evaluate the magnitude and quality of OVA-specific CD8+ T cell response by intracellular cytokine staining (ICS). A representative experiment is shown. CD8+ T cells were analyzed in splenocytes stimulated with OVA 8mer peptide (OVApep). The percentage of CD8+ T cells producing IFNγ and TNFα and expressing CD107a is indicated within the quadrants.

To validate the efficacy of IDLV as anti-tumor vaccine in an immunotherapeutic approach, we initially selected the E.G7-OVA lymphoma model. Fourteen C57BL6 mice were injected s.c. with 3.5 × 10^6^ E.G7-OVA/mouse (Figure 1a). At day 6, when the tumor reached 10 mm of diameter, a group of seven mice was immunized once i.m. with IDLV-OVA (3 × 10^6^ RT units/mouse). Tumor growth was monitored over time. All control mice were sacrificed within 18 days from tumor injection. Results showed that a single immunization was able to eradicate the tumors in all animals (Figure 1b). One out of seven vaccinated mice developed tumors at 60 days and was sacrificed, while the other vaccinated mice remained tumor free (Figure 1c). To evaluate the persistence of the protective vaccine-induced immune response, 175 days after the first tumor injection, 3.5 × 10^6^ E.G7-OVA cells were administered s.c. in the surviving and tumor-free mice (Figure 1d). None of the vaccinated mice challenged with a second inoculation of tumor cells developed tumors as opposed to control mice that died within 24 days. Vaccinated mice maintained a tumor-free status until the end of the experiment (125 days after the second tumor injection, 300 days total).

The OVA-specific immunity induced by IDLV-OVA vaccination was measured throughout the entire period of the experiment (Figure 1e). All vaccinated mice showed OVA-specific CD8+ T cells, evaluated by IFNγ ELISpot assay, starting from 8 days after immunization, corresponding to 14 days after tumor injection (837 ± 167 spot forming cells (SFC)/10^6^ cells, mean ± SD) reaching a peak at 35 days (1064 ± 293 SFC/10^6^ cells). None of the control mice showed OVA-specific response after E.G7-OVA injection (data not shown). The response was persistent, showing an average value of 644 ± 284 SFC/10^6^ cells at 104 days from tumor injection. After the second tumor injection, the OVA-specific response increased and persisted at high level up to 300 days from the single immunization (734 ± 151 SFC/10^6^ cells). At sacrifice, splenocytes were analyzed for the presence of OVA-specific polyfunctional effector CD8+ T cells (IFNγ+/TNFα+/CD107a+), by intracellular cytokine staining (ICS). The lysosomal-associated membrane proteins (LAMPs or CD107a) appear on the cell surface after cytotoxic granules exocytosis, as functional markers of cytotoxic activity. A representative experiment performed on splenocytes from an immunized mouse is shown in Figure 1e. Splenocytes were stimulated with OVA 8mer peptide or left untreated and then analyzed for CD107a expression on the membrane and the intracellular IFNγ and TNFα expression by gating on CD3+/CD8+ T lymphocytes (Figure 1f). Only upon OVA-stimulation, CD8+ T cells from immunized mice express all the markers analyzed, confirming their cytotoxic profile.

Altogether, these results show that a single immunization with IDLV-OVA induced a strong, persistent, and functional CD8+ T cell-mediated OVA-specific response able to eradicate a preexisting large tumor mass as well as to generate memory cells able to prevent potential relapses.

### 3.2. IDLV-OVA Vaccination Controlled B16OVA Tumor Growth and Survival in Both Preventive and Therapeutic Settings

Since a single immunization with IDLV-OVA was able to eradicate large E.G7-OVA tumor masses, we decided to use the more aggressive B16 melanoma model, a spontaneous melanoma derived from C57BL/6 mice, used as tumor model for preclinical evaluation of immunotherapies. B16OVA derives from parental B16 cell line after stable transfection with a plasmid expressing OVA. The expression of OVA was confirmed by RT-PCR (data not shown). We initially investigated the ability of the long-term vaccine-induced memory OVA-specific CD8+ T cells to prevent the in vivo B16OVA growth in a preventive experimental setting, as partially already published [31]. Briefly, mice immunized either with IDLV-OVA or IDLV expressing an unrelated antigen (IDLV-Mock), or left untreated (Naïve), were injected s.c. with 2 × 10^5^ B16OVA cells at 26 weeks after the single immunization. Four out of six IDLV-OVA vaccinated mice developed tumors with a marked and significant delay, while two out of six mice remained tumor-free until the end of the study [30]. OVA-specific immune response was monitored over time by IFNγ ELISpot assay. As expected, the anti-OVA immune response (Table 1) was strong in the IDLV-OVA immunized mice until 26 weeks after immunization, showing a large number of OVA-specific T cells producing IFNγ. We confirmed the strong and sustained anti-OVA immune response in all IDLV-OVA vaccinated mice, until 19 weeks from tumor injection, evaluated in the tumor-free remaining mice.

**Figure 2:**
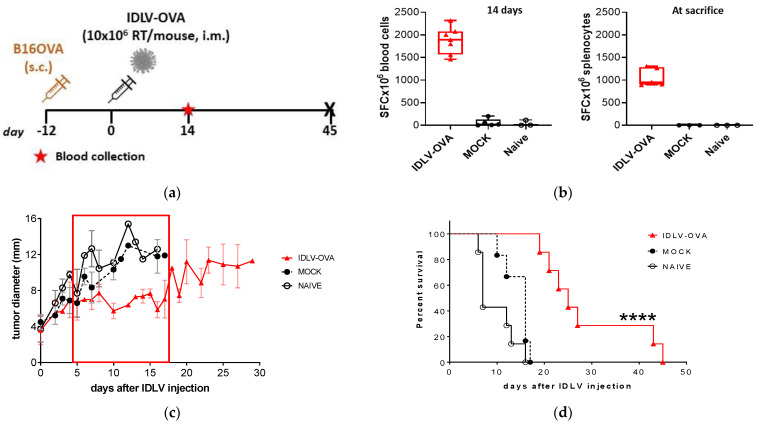
Therapeutic efficacy of IDLV-OVA vaccination in B16OVA-bearing mice. (**a**) Scheme of the experiment. C57BL/6 mice were s.c. injected with 2 × 10^5^ B16OVA cells/mouse. After 12 days, groups of mice were vaccinated either with 10 × 10^6^ RT/mouse of IDLV-OVA or IDLV expressing an unrelated antigen (mock) or left untreated (Naïve). (**b**) OVA-specific T cell response was generated in all IDLV-OVA immunized mice, as evaluated by IFNγ ELISpot measured two weeks after immunization in blood cells (left panel) and at sacrifice in splenocytes (right panel). Cells were collected and stimulated with H-2Kb restricted OVA 8mer peptide (SIINFEKL). Data are expressed as spot forming cells (SFC) per million cells. (**c**) Tumor growth. All groups developed a tumor mass measured until the end of the experiment. The delay of tumor growth in IDLV-OVA immunized mice is highlighted with the red rectangle. Mock and Naïve groups were sacrificed within 18 days from tumor injection. (**d**) Kaplan–Meier survival curve. Mice with tumor diameter >15 mm or a serious ulceration were sacrificed. (Log-rank Mantel-Cox test, **** *p* < 0.0001).

To assess the therapeutic efficacy of IDLV-OVA in B16OVA model, three groups of mice were inoculated s.c. with 2 × 10^5^ B16OVA and when tumor mass was palpable, mice were vaccinated with 10 × 10^6^ RT units/mouse of IDLV-OVA, IDLV-Mock, or left untreated, following a protocol schedule depicted in Figure 2a. Fourteen days after immunization blood samples were collected to perform IFNγ ELISpot assay. All IDLV-OVA treated mice showed high levels of OVA-specific response (1873 ± 296 mean SFC/10^6^ cells), very low or undetectable in control mice (57 ± 84 and 40 ± 63 in Mock and Naïve groups, respectively) (Figure 2b). In all naïve and mock-injected mice, the tumor continued to grow, and all control mice were sacrificed within 18 days (Figure 2c,d). On the contrary, all IDLV-OVA vaccinated mice showed a significant control of tumor growth compared with control groups (Figure 2c). Tumors started to grow again 20 days after immunization and all vaccinated mice died within 45 days from the immunization (Figure 2d). These data indicate that a single immunization with IDLV-OVA was able to induce a significant delay of a very aggressive melanoma growth and a significant increase in survival (*p* < 0.0001).

**Figure 3:**
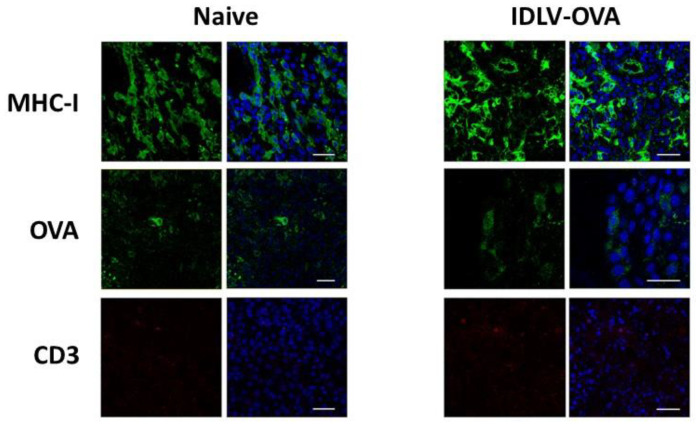
Confocal laser scanning microscopy (CLSM) analyses on mice tissue sections. Representative images of tumor from Naïve and IDLV-OVA mice are shown. Tissue sections 8 µm thick were stained for MHC Class I (green), OVA (green), or CD3 (red) as indicated (left columns) and for DAPI as nuclear staining (blue, right columns). Images represent a 3D reconstruction of 30–40 single Z-stack. Results from one representative experiment are shown for each analysis. Scale bars are 40 µm.

To assess the expression pattern at the tumor site, MHC Class I (MHC-I), CD3, and OVA expression was assessed by confocal microscopy in tumors collected at sacrifice. As shown in Figure 3, MHC-I is highly expressed in vivo in tumor cells, while OVA-expressing cells are barely detectable in both naïve and immunized mice. T cell infiltration, measured as CD3+ cells, is very low. These results suggest that low expression of the tumor-specific antigen OVA together with poor T cell infiltration could have affected the therapeutic efficacy of the vaccination.

### 3.3. Self-TAA in the Melanoma Model

**Figure 4:**
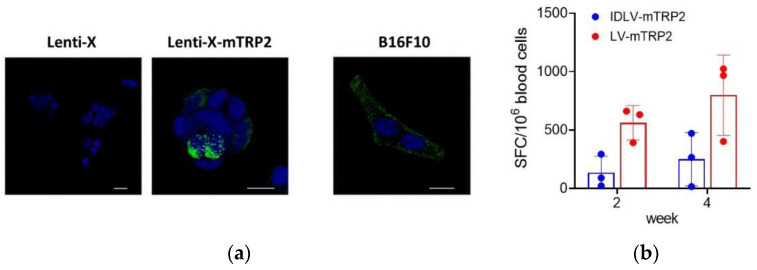
IDLV expressing mTRP2 as a self-antigen induces low specific T cells. (**a**) Expression of mTRP2 in cell lines. Mock Lenti-X cells, pTY2CMVmTRP2W-transfected Lenti-X cells and B16F10 cells were fixed and stained with anti-TRP2 antibody (green) and DAPI (blue) and analyzed by CLSM. Images represent a 3D reconstruction from 14 single optical sections. Results from one representative experiment are shown for each analysis. Scale bars are shown for each figure. (**b**) Pilot study of immunogenicity. C57Bl6 mice (*n* = 3) were immunized once with 10 × 10^6^ RT units/mouse of either IDLV-mTRP2 or LV-mTRP2. The TRP2-specific T cell-mediated immune response was evaluated by IFNγ-ELISpot assay at two and four weeks in blood. Blood cells were stimulated with H-2Kb restricted TRP2 9mer peptide (SVYDFFVWL). Data are expressed as specific spot forming cells (SFC) per million cells. Box plots show mean ± SEM and single values from each immunized mouse.

TRP2, expressed by most human melanoma cells, represents an attractive target for melanoma vaccines. The murine tumor cell line B16F10 is an aggressive clone derived from B16 cells and expresses the murine (m) homologous of human (h) TRP2. To verify the expression of the codon optimized mTRP2 generated for the production of lentiviral vectors used in this study, Lenti-X cells were transfected with the plasmid expressing mTRP2 and analyzed by confocal microscope, using B16F10 as a positive control. The images confirmed consistent expression in both B16F10 and transfected LentiX cells (Figure 4a).

Breaking tolerance to a self-antigen is an important challenge to be considered in order to induce an efficient anti-tumor immune response. We performed a pilot study of immunogenicity in mice to measure the TRP2-specific T cell immune response induced after a single immunization with IDLV-mTRP2. Based on our previous dose-response experiments, using LV and IDLV delivering different antigens [26,31,33], we decided to directly inject 10 × 10^6^ RT/units/mouse, as a selected dose able to efficiently induce strong T cell immunity. A group of mice immunized with the same dose of integrase competent LV-mTRP2 was included as a positive control of vaccine-induced immune response. As shown in Figure 4b, the T cell response was low in 2 mice and negative in 1 mouse in the IDLV group, while LV-immunized mice showed a higher response, as expected. In both groups, the response increased from two to four weeks after immunization. These data indicate that high expression of the self-antigen is needed to break tolerance in vivo.

**Figure 5:**
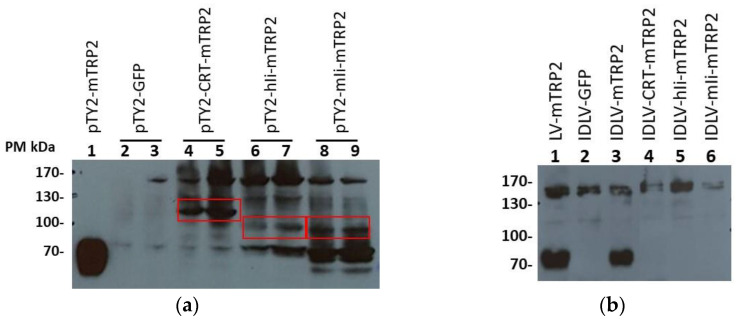
Expression of different TRP2 fusion proteins by Western blot. (**a**) Cell lysates of Lenti-X transfected with transfer vector expressing either mTRP2 (predicted size 70 kDa) or mTRP2 fused with CRT (Calreticulin, predicted size 123 kDa), hIi (human invariant chain, predicted size 109 kDa), or mIi (murine invariant chain, predicted size 106 kDa). Lenti-X cells transfected with mTRP2 wild type (5 × 10^4^ cells, lane 1) and GFP transfer vectors (lanes 2 and 3) were used as positive and negative control, respectively. The assay was performed using 1.6 × 10^5^ (lane 2–4–6–8) or 3.2 × 10^5^ cells (lane 3–5–7–9). Red boxes indicate the band with the correct molecular weight. (**b**) Detection of mTRP2 in viral vector preparations. 1.5 × 10^6^ RT units of LV-mTRP2 (lane 1), IDLV-GFP (lane 2), IDLV-mTRP2 (lane 3), IDLV-CRT-mTRP2 (lane 4), IDLV-hIi-mTRP2 (lane 5), IDLV-mIi-mTRP2 (lane 6) were analyzed.

To improve the IDLV-mTRP2 efficacy in terms of induction of T cell response, we decided to fuse the mTRP2 transgene with protein/peptide sequences known to improve the immunogenicity. Among these, the major histocompatibility complex class II–associated invariant chain (Ii) [34] and Calreticulin (CRT) [29] have been shown to improve the induction of CD8+ T cells when fused with a non-self-antigen. CRT, murin (m)Ii and human (h)Ii were used to produce the modified mTRP2 fusion proteins (CRT-mTRP2, miI-mTRP2, and hiI-mTRP2) and expressed from transfer vectors (Figure S1) to generate IDLVs that we expected to improve the antigen-specific immune response. To verify the expression of the modified TRP2 proteins, Lenti-X cells were transfected with the three different plasmids. As shown in Figure 5a, specific bands corresponding to the predicted molecular weights were detected. IDLV delivering the three modified transgenes were produced and used to immunize mice. IFNγ-ELISpot assay was performed at 2 and 8 weeks from immunization in blood cells. Surprisingly, no specific TRP2-specific T cell-mediated immune response was detected in all groups of mice vaccinated with modified TRP2 antigens (data not shown). We hypothesize that the modifications interfered with the expression, folding or intracellular pathway of the modified transgene. Since TRP2 is a transmembrane protein, we evaluated the presence of TRP2 in the vector preparations used to immunize mice, by Western blot (Figure 5b). TRP2 was detected in LV-mTRP2 and IDLV-mTRP2, while the fusion proteins were not present in the relative IDLV preparations. Finally, to exclude a problem in delivering the transgene by the vector, expression of TRP2 fusion proteins was analyzed by Western blot in Lenti-X transduced with vectors. As shown in Figure S2, the bands corresponding to the fusion proteins were present, although at much lower levels compared to the band observed in the cells infected with the vector encoding the wild type TRP2. Altogether, these results suggest that undetectable TRP2-specific T cell response was likely due to fusion protein altered expression.

### 3.4. The human TRP2 as an Alternative Antigen Together with an Increase of Dose Resulted in a Better Efficacy of IDLV Vaccination

**Figure 6:**
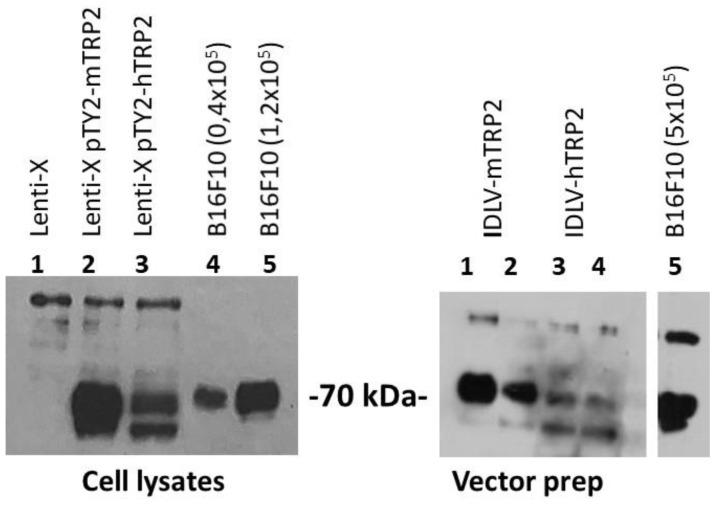
Expression of murine and human TRP2 evaluated by Western blot. Left panel: Detection of TRP2 in cell lysates of Lenti-X cells (1 × 10^5^) transfected with plasmids expressing either mTRP2 or hTRP2. Lenti-X and B16F10 cell lysates were used as negative and positive control of TRP2 expression, respectively. Right panel: Detection of TRP2 in concentrated vector preparations (3 × 10^6^ RT/lane). The rabbit anti-TRP2 polyclonal antibody used to detect the expression of TRP2 recognizes both human and murine TRP2 proteins.

Human TRP2 (hTRP2) represents the human counterpart of murine TRP2 used in previous experiments and shares the immunodominant peptide (SVYDFFVWL) recognized by T cells in the context of HLA-A201 with the murine H2-Kb-restricted epitope in C57BL6 mice. We investigated whether immunization with IDLV delivering hTRP2, which is 80% identical with mTRP2 at the protein level, would be more effective in breaking tolerance against the self-protein TRP2, as previously described [35]. The expression of hTRP2 was confirmed by Western blot in Lenti-X trasfected with pTY2-hTRP2 plasmid (Figure 6, left panel) or concentrated vector preparations of IDLV delivering either murine or human TRP2 (Figure 6, right panel).

**Figure 7:**
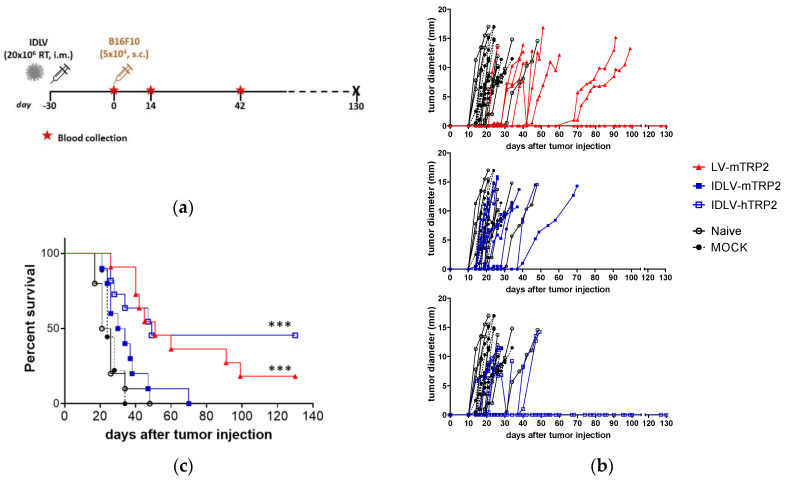
Antitumor efficacy of lentiviral vectors expressing either murine or human TRP2. (**a**) Scheme of the experiment. C57BL/6 mice (*n* = 9–11) were immunized with vectors expressing TRP2, OVA (Mock) or left untreated. After 30 days all mice were s.c. injected with 5 × 10^4^ B16F10 cells/mouse. (**b**) Tumor growth was monitored over time. All groups developed a tumor mass measured until the end of the experiment. Mock and Naïve groups were sacrificed within 32 days from tumor injection. (**c**) Survival was monitored over time and Kaplan–Meier survival curve is shown. Mice with tumor diameter >15 mm or a serious ulceration were sacrificed. (Log-rank Mantel-Cox test, *** *p* < 0.0001).

Groups of 9–11 mice were i.m. immunized once with high dose of IDLVs expressing either human or murine TRP2, according to the scheme depicted in Figure 7a. LV-mTRP2 was used as a positive control, while IDLV-OVA (Mock) and Naïve mice were included as negative controls for immunization and for tumor growth. Blood samples were collected at the day of tumor injection (day 0, corresponding to 30 days after immunization), at 14 and 42 days after tumor injection.

Tumor developed in mock and Naive groups and all but one control mouse were sacrificed within 34 days from tumor injection (Figure 7b,c). All TRP2 vaccinated mice showed a delay in tumor growth (Figure 7b). IDLV-mTRP2 showed a delay of tumor growth, but all mice died by 70 days after tumor injection, while a significant delay was observed in mice vaccinated with LV-mTRP2 and 18% of mice remained tumor free until the end of the experiment (130 days), suggesting that high levels of transgene expression is able to break tolerance efficiently and prevent partially tumor growth. Interestingly, IDLV delivering the human version of TRP2 was able to delay tumor growth and increased significantly survival compared to control mice (Figure 7c). Of note, 45% of mice remained tumor free until the end of the experiment.

**Figure 8:**
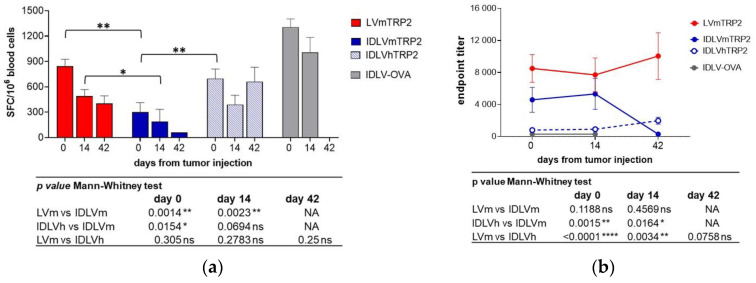
Kinetics of TRP2-specific immune responses in mice immunized with IDLV delivering either murine or human TRP2 and challenged with B16F10 tumor. C57BL/6 mice (*n* = 9–11) immunized with vectors expressing TRP2, OVA, or left untreated were s.c. injected with 5 × 10^4^ B16F10 cells/mouse after 30 days from immunization, as depicted in Figure 7a. (**a**) TRP2-specific T cell response was evaluated by IFNγ ELISPOT, measured at different time points in blood. Cells were collected and stimulated with H-2Kb restricted TRP2 9mer peptide (SVYDFFVWL). Data are expressed as mean spot forming cells (SFC) per 10^6^ cells, bars represent standard error among animals from the same group. (**b**) Anti-mTRP2 IgG antibodies were analyzed in plasma of immunized animals by ELISA. Data are expressed as mean of endpoint titers and bars represent standard error among animals from the same group. Comparison among groups was evaluated using the Mann–Whitney test, as indicated by *p* values shown in the tables under the graphs. * *p* < 0.05; ** *p* < 0.01; **** *p* < 0.0001. NA: The comparison was not performed since only one animal from IDLVmTRP2 group was alive at day 42.

All groups of vaccinated mice showed a specific T cell response after the immunization, measured by IFNγ ELISpot (Figure 8a). The magnitude of response was different in the different groups. In particular, LV-mTRP2 and IDLV-hTRP2 induced significantly higher TRP2-specific T cell response compared to IDLV-mTRP2 immunized mice that showed the lowest induction of IFNγ producing T cells at the indicated time points, as specified in the table below the graph in Figure 8a. As expected, mice injected with the mock vector (IDLV-OVA) showed strong OVA-specific (1307 ± 96 SFC/10^6^, Figure 8a) but not anti-TRP2 immune response (data not shown). Naive mice did not show antigen-specific T cell responses (data not shown). The IFNγ ELISpot performed at 42 weeks after tumor injection in those mice that were still alive strongly suggests that the magnitude of TRP2-specific T cell response correlates with the survival increase (663, 403 and 62 mean SFC/10^6^ cells in IDLV-hTRP2, LV-mTRP2, and IDLV-mTRP2, respectively). TRP2 as a membrane protein can be considered a target of antibody-mediated cytotoxic activity (ADCC). Therefore, we assessed the presence of anti-mTRP2 IgG in the plasma of all immunized animals before and after tumor injection. Figure 8b shows the antibody titers over time, measured by ELISA. All TRP2-vaccinated mice showed anti-mTRP2 IgG, although at different titers. In particular, immunization with LV and IDLV expressing mTRP2 induced significantly higher titers than IDLV-hTRP2 at the indicated time points (Figure 8b, table under the graph). As expected, specific anti-TRP2 antibody titers were not detected in Mock control mice immunized with IDLV-OVA.

**Figure 9:**
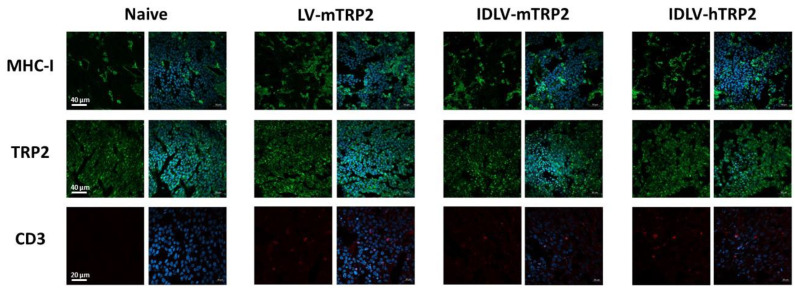
Confocal laser scanning microscopy (CLSM) analyses on mice tissue sections. Representative images of tumor from naïve, LV-mTRP2, IDLV-mTRP2, and IDLV-hTRP2 mice are shown. Tissue sections 8 µm thick were stained for MHC–I (green), TRP2 (green), or CD3 (red) as indicated (left columns) and for DAPI as nuclear staining (blue, right columns). Images represent a 3D reconstruction of 30–40 single Z-stack. Results from one representative experiment are shown for each analysis. Scale bars are indicated.

At sacrifice, the tumor mass was analyzed by confocal microscope (Figure 9) for the expression of relevant markers. High expression of both MHC-I and mTRP2, but few infiltrating T cells were detected in TRP2 immunized mice, regardless of the vaccine regimen.

## 4. Discussion

Several groups have investigated and progressively improved different immunotherapeutic strategies, to induce high-quality and heterogenic immune responses able to eradicate pre-existing tumor mass as well as to avoid their progression or possible relapses [36,37].

In this study, we analyzed the potential efficacy of IDLV pseudotyped with VSV.G as a delivery system of TAAs in preventing and controlling tumor growth and increasing survival of tumor bearing mice. We and others previously demonstrated the strong efficiency of IDLV in inducing a potent and protective T cell immunity in different infectious disease models, including the HPV16 tumor model [29]. Here, we further validated the IDLV-based platform for cancer immunotherapy, using different tumor models, starting from a non-self-TAA (OVA) as selected transgene, using two syngeneic tumor cell lines, E.G7-OVA and B16OVA. The E.G7-OVA tumor cell line is highly immunogenic and widely used in the immunology field both in vitro as target cells in cytotoxicity assays and in vivo in mice as a model of antigen specific immunotherapy [38], a relatively simple tumor model to eradicate in vivo through the optimal induction of OVA-specific CTL [39]. A single immunization with IDLV-OVA was indeed able to eradicate large tumor masses in E.G7-OVA tumor bearing mice. The analysis of OVA-specific T cell response induced by vaccination revealed a high number of functional and persistent CTL. The presence of these effector cells allowed a complete rejection of a second challenge, performed at six months after the immunization, confirming the protective immunity conferred by IDLV-OVA vaccination.

Subsequently, we investigated the efficacy of IDLV-OVA immunization in a more difficult tumor model, such as B16OVA, a melanoma murine cell line expressing OVA derived from parental B16 cell line [10,31]. We initially investigated the ability of IDLV-induced immunity to prevent B16OVA growth, injecting the tumor 26 weeks after IDLV-OVA immunization, during a memory phase of OVA-specific response. A statistically significant increase of survival as well as a delay of tumor growth compared with control mice was shown with about 30% of vaccinated mice remaining tumor free [30]. In this report, we evaluated an immunotherapeutic strategy by immunizing B16OVA-bearing mice. In this setting, IDLV-OVA immunized mice showed delayed tumor growth and increased survival compared to control mice, although the vaccination did not eradicate the tumor mass. At tumor level, the low number of OVA-expressing tumor cells may in part explain the partial therapeutic efficacy observed. Further investigation at the tumor site should be pursued to understand better the role of the microenvironment on the T cell activity and functionality. Overall, these data suggest that a single immunization with IDLV delivering a non-self TAA has the concrete potential to counteract growth of different tumors. Our data are in line with those obtained by using LV305, an IDLV targeting dendritic cells and delivering NY-ESO1 as non-self TAA in mice [40].

In a more difficult setting, we also tested the ability of IDLV expressing a self-TAA to break tolerance and induce an immune response able to prevent the growth of B16F10 tumor. Low immune response induced by IDLV-mTRP2 vaccination suggested that a higher expression of transgene associated with a more efficient MHC-I presentation was necessary. Indeed, we demonstrated that LV-mTRP2 was able to break tolerance more efficiently, as shown by the B16F10 growth control.

In an effort to improve TRP2 immunogenicity, we generated IDLV delivering mTRP2 protein fused to different “molecular enhancers”, including CRT, mIi, and hIi, previously shown to improve immunogenicity towards non-self-antigens [29,34]. However, none of these new IDLVs induced detectable immune response in vivo. We hypothesize that the low expression and/or interference in folding or in the intracellular pathway of the TRP2 fused to the molecular enhancer resulted in the breakdown of immune response. Alternatively, presence of TRP2 protein on IDLV particles may be necessary for induction of effective immune response. Indeed, while the wild type murine and human TRP2 proteins were assembled on the lentiviral particle as transmembrane protein, the enhancer-modified TRP2 were not detected.

The TRP2 protein detected in the vector preparations (Figure 5b and Figure 6) could also be derived from pseudotyped vector particles and extracellular vesicles, such as exosomes. As protein carriers, these particles can contribute to induce antigen-specific response after in vivo injection. In a previous study, using Influenza hemagglutinin (HA) as a different model antigen, we showed that while pseudotyping the lentiviral particles with HA induced strong and functional humoral responses against the pseudotyping protein, the expression of the same HA as a transgene from IDLV also induced T cell responses [41]. Moreover, in other studies using soluble HIV-1 Envelope (HIV-Env) and GFP as model antigens, we produced control IDLV expressing a non-relevant transfer vector in cells that expressed the antigen ectopically from a plasmid that cannot be packaged into the vector [26,27]. Results showed the absence of any antigen-specific responses, indicating that protein contaminating the IDLV preparation due to pseudo-transduction, if any, did not contribute to the induction of antigen-specific immunity. Although we cannot exclude the role of exosomes and pseudotyped particles in the induction of TRP2-specific immune response after IDLV-TRP2 injection, our previous work strongly indicates that the transgene is a major player of the IDLV-induced T cell immunity.

Finally, we improved the immunity to TRP2 by using human instead of murine TRP2. Previous work has shown that immunization with xenogeneic, evolutionarily conserved antigens or modified self-proteins is able to break tolerance to the corresponding self-antigens [42,43]. In our study, IDLV-hTRP2 induced a superior T cell response compared to IDLV-mTRP2, resulting in a significant increase of survival of tumor-bearing mice and preventing tumor growth. It has been reported that the efficacy of a vaccine based on alphavirus replicon particles (VRP) expressing a melanoma antigen, relies on its ability to elicit specific antibodies that synergistically co-operate to tumor protection together with CD8+ T cells with a mechanism involving CD16 [44]. This approach is substantially different from our platform, being based on the in vivo replication of mRNA, which results in a strong expression of the antigen. In our study, TRP2-specific T cell response, but not anti-mTRP2 IgG antibodies, seemed to correlate with tumor control and survival increase. Although tumor cells expressed high level of TRP2 and MHC-I in vivo, T cell infiltration was very poor, impairing the potential effectiveness of CTL to kill TAA-expressing cells.

Overall, these data indicate that the IDLV-based platform is a suitable and efficient strategy for cancer immunotherapy. A single immunization of IDLV induced a functional anti-tumor immunity able to prevent tumor growth, increased survival of tumor-bearing mice or completely eradicated established tumors, depending on the tumor model used and the experimental setting. These data strongly suggest that this platform should be further exploited and optimized. To this regard, the use of combined approaches should be investigated, including the use of checkpoint inhibitors [45] and administration of drugs known to increase the transgene expression from IDLV episomes, such as histone deacetylase inhibitors (HDACi) [46,47] or chemotherapeutic drugs that stimulate the immune response [48].

## Figures and Tables

**Table 1:** Kinetics of OVA-specific T cell immune response evaluated in mice immunized with IDLV-OVA and injected with B16OVA by IFNγ ELISpot.

Weeks after Immunization	Mean (SFC/10^6^ Cells)	±SD
2	957	28
8	1020	255
26 (tumor injection)	947	98
32 (6 after tumor injection)	1037	288
45 (19 after tumor injection)	774	212

## Data Availability

Data is contained within the article and supplementary material.

## Supplementary Materials

The following are available online at https://www.mdpi.com/1999-4915/13/2/355/s1, Figure S1: Schematic representation of self-inactivating (SIN) transfer vector and pU57 plasmids used in this study. Figure S2: TRP2 expression in Lenti-X transduced with vectors expressing TRP2 fusion proteins.
