# Supplementary material for: Integrase-Defective Lentiviral Vector Is an Efficient Vaccine Platform for Cancer Immunotherapy
Source: Viruses. 2021 Feb 23;13(2):355. doi: 10.3390/v13020355 (PMC7927015; doi:10.3390/v13020355)
Supplement: Supplementary file 1 [file viruses-13-00355-s001.pdf]

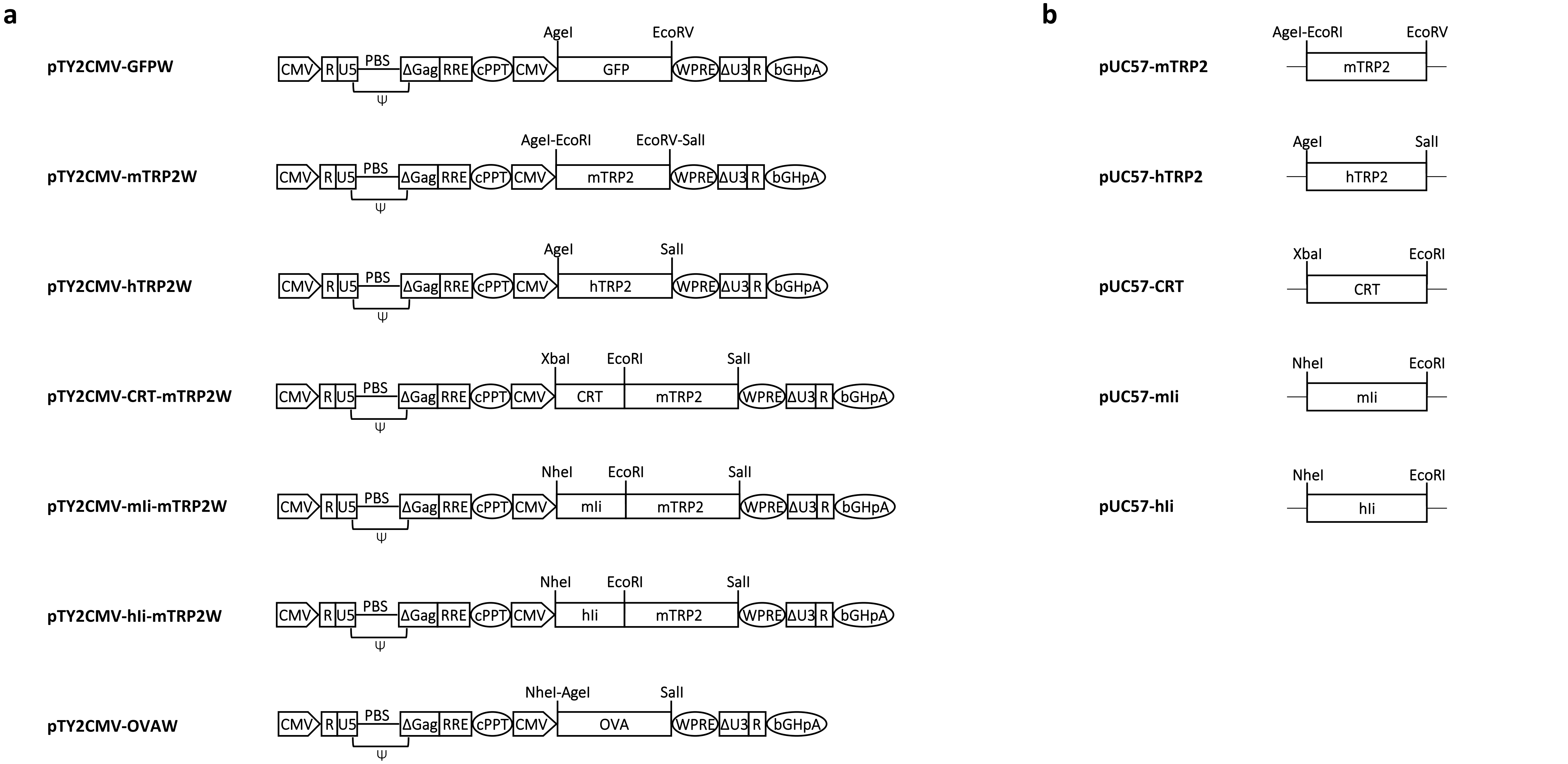

Figure S1. Schematic representation of self-inactivating (SIN) transfer vector and pU57 plasmids used in this study. **(a)** Transfer vectors expressing GFP, mTRP2, hTRP2, CRT-mTRP2, mIi-mTRP2, hIi-mTRP2, and OVA from the internal CMV promoter. **(b)** Codon optimized mTRP2, hTRP2, CRT, mIi, and hIi sequences in pUC57 plasmids. CMV, cytomegalovirus immediate-early promoter; R, repeat element; U5, 5’ untranslated region; U3, 3’ untranslated region; PBS, primer binding site; Ψ, packaging signal; cPPT, central polypurine tract; RRE, Rev response element; ΔU3, SIN deletion in U3 region of 3’ LTR; WPRE, woodchuck hepatitis virus post-transcriptional regulatory element; bGHpA, bovine growth hormone polyadenylation signal. See Methods for details on construction.

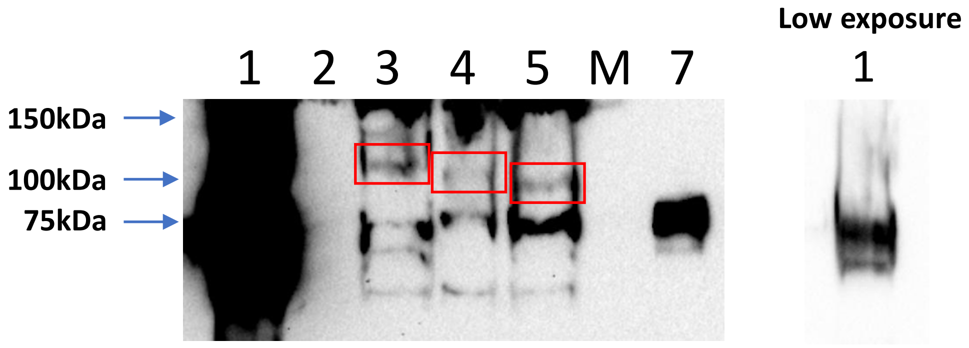

Figure S2. **TRP2 expression in Lenti-X transduced with vectors expressing TRP2 fusion proteins.** Left: Western blot of cell lysates ($3x10^6$ cells) of Lenti-X transduced with lentiviral vectors expressing either mTRP2 (predicted size 70 kDa, lane 1) or mTRP2 fused with CRT (Calreticulin, predicted size 123 kDa, lane 3), hIi (human invariant chain, predicted size 109 kDa, lane 4) or mIi (murine invariant chain, predicted size 106 kDa, lane 5), and GFP (lane 2), used as negative control. B16F10 cells ($25x10^4$, lane 7) were used as a positive control. M: Markers. Right: A lower intensity exposure of the gel to better visualize mTRP2 in Lane 1.
